# The Pathogenesis of Pyrogen

**Published:** 1893-08

**Authors:** W. A. Yingling

**Affiliations:** Nonchalanta, Kan.


### THE PATHOGENSIS OF PYROGEN.

NONCHALANTA, KAN., July 12th, 1893.

EDITOR HOMŒOPATHIC PHYSICIAN:

I send you herewith the MS. of *Pyrogen*. You will find it to be reliable, as I was very careful not to use any symptom where other remedies were used in connection with this one. I wished to make it reliable so that all could use it with assurance.

It is not as long an article as it seems, as I have used space so as to make it plainer to the printer. I wish you would print it, if at all acceptable to you, as soon as you can, so the profession can get to using it. I am sure very many will thank you for the pathogenesis of a remedy so highly thought of at the present time. It is a grand remedy. The one clinical case I report from my own practice would make it of much interest. Whilst this paper has cost me much time, thought, and work, yet I shall be fully repaid if others can use Pyrogen to good advantage.

I would like to make out reports of other remedies for you, but I have no data that are reliable, as all the remedies used by so many are alternated, which precludes any reliance being placed in them. Still, I guess this fulfills my promise to you to report some of the new remedies. I shall not forget to keep an eye out for anything else that may be of interest to your readers. I like to do good and to be of some use to the profession.

Fraternally,

W. A. YINGLING.

